# Report of a Case of Diseased Ear, Which Produced Death in a Child of Four Years of Age

**Published:** 1843-04

**Authors:** Thomas Carroll

**Affiliations:** Cincinnati


*Report of a case of Diseased Ear, which produced death, in a child of four years of age.*—By Thomas Carroll, M. D., of Cincinnati.—M. W., a female child, was admitted into the Orphan Asylum, at the age of two years, and at the time of admission was laboring under chronic diarrhœa, which had worn it down to the last degree that life could endure; and at the same time it had suppuration of the right ear. After being in the Asylum some months, its health became better; but the ear continued to run, and the abdomen enlarged to unusual size. Notwithstanding the improvement of the general health, the diarrhœa continued, and the ear gave no evidence that suppuration would cease, though the diarrhœa at times seemed to promise a total cessation, yet it returned and became more severe at short intervals, until death. The strength did not improve as was expected, at least as far as regarded the inferior extremities, which latter circumstance was probably owing to the want of care, in not making her use them in endeavoring to stand. The want of proper exercise of the lower extremities caused a great disproportion in size between them and the rest of the body.

When I first enquired into the situation of this patient, which was in March, 1842, I found that she was fretful, and seemed to suffer pain, as she spent large portions of her nights in sitting up in her cradle and rocking herself con stantly for hours; and afterwards would sleep much through the day.

As the ear ran very much, I directed a solution of the nitrate of silver to be injected into it several times a day, which was sometimes to be intermitted, and suds or warm water to be used in its place. Blisters were applied behind the ears, and her diet was rigidly regulated. For the derangement of the bowels one grain of Dover's powder, one of rhubarb, and half a grain of calomel, were combined and occasionally given. About a month before her death, a phlegmonous tumor appeared on the right hip, and suppurated, and discharged considerable matter. No sooner did this abscess heal, than the ear seemed more aggravated; the diarrhœa increased, and the whole side of the head became very much swollen; and the motory nerves on the side of the face lost their influence, showing that the portio dura of that side had become diseased. But, if motion, which had been given by this nerve, was lost; sensation, that in this part is derived from the fifth pair, was most acute. We are told by Sir C. Bell, that when a diseased condition, or rather destruction, of the expansion of the seventh pair of nerves on the face takes place, that the eye-lids of the affected side cannot be closed, as the orbicularis palpebrarum has its motory influence from that source. Aside from this, the present case is a beautiful illustration of the principles of Bell, for the child had the power of shutting the eye on the affected side.

The matter discharged from the ear had long been offensive, and it was evident that caries of the petrous portion of the temporal bone had existed to some extent for many months, but now the evidences were more decided, as the cavity of the ear received a larger amount of fluid than usual; and from examination the tympanum appeared to be gone. Large amounts of pus of an ill-conditioned kind was constantly discharged. The inflammation became intense over the mastoid process, and anterior of the ear; and forty-eight hours before death mortification of the integuments over the mastoid process took place; a few hours after a similar condition resulted anterior to the ear; and at death the ear was nearly ready to drop off. Poultices alone seemed to give any relief.

During the last few days the patient showed evident symptoms of great pain in the head; but at no time was there the least evidence of delirium, though much irritability of temper was evinced to the last moments of life.

After death I examined the morbid appearances of the head, aided by Drs. Woodward, Warder and Young. In exposing the cranium in the usual way, the cavity around the ear was laid open, and was found to contain considerable pus. The cavity extended over most of the squamous and petrous portions of the temporal bone, followed the zygomatic process nearly to the malar bone, and extended down the condyloid process of the maxillary bone to its connection with the angle of the jaw. The bones had lost their proper covering every where connected with the abscess; even the internal ear had not a vestage of any tissue covering the bone; and its inferior wall was perforated by caries about the eighth of an inch from the outer margin, which had been itself in a carious state for a considerable time. The foramen for the entrance of the internal carotid artery had thrown off its proper investment, and the artery meandered naked through it. The portio dura was either totally or nearly destroyed at its exit.

On the inner side of the temporal bone, and immediately at the entrance of the internal carotid, the dura mater was found inflamed, and separated from the bone. At the base of the cerebellum the arachnoid membrane was opaque, and the surface of the brain abnormally vascular. About two ounces of fluid escaped from the surface and ventricles when the brain was exposed.

The pupils of the eye in this patient never dilated, and the eyes continued clear all the time—an instructive lesson with regard to the symptoms indicating effusion within the cranium.

This patient died at the age of four years, and had been under my care from the middle of February, 1842, until August of the same year. I do not know that any treatment could have been instituted, at any time, that would have proved efficacious in the management of the case; it is most probable that nothing could have been done, unless at a very early period. I have no knowledge of the circumstance that led to the suppuration of the ear, nor do I know at what time of life it took place.—*Western Lancet.*

